# Data accessibility in the chemical sciences: an analysis of recent practice in organic chemistry journals

**DOI:** 10.3762/bjoc.21.70

**Published:** 2025-05-02

**Authors:** Sally Bloodworth, Cerys Willoughby, Simon J Coles

**Affiliations:** 1 School of Chemistry and Chemical Engineering, University of Southampton, Highfield, Southampton SO17 1BJ, UK

**Keywords:** data availability, FAIR principles, journal guidelines, NMR data, organic chemistry

## Abstract

The discoverability and reusability of data is critical for machine learning to drive new discovery in the chemical sciences, and the ‘FAIR Guiding Principles for scientific data management and stewardship’ provide a measurable set of guidelines that can be used to ensure the accessibility of reusable data. We investigate the data practice of researchers publishing in specialist organic chemistry journals, by analyzing the outputs of 240 randomly selected research papers from 12 top-ranked journals published in early 2023. We investigate compliance with recommended (but not compulsory) data policies, assess the accessibility and reusability of data, and if the existence of specific recommendations for publishing NMR data by some journals supports author compliance. We find that, although authors meet mandated requirements, there is very limited compliance with data sharing policies that are only recommended by journals. Overall, there is little evidence to suggest that authors’ publishing practice meets FAIR data guidance. We suggest first steps that researchers can take to move towards a positive culture of data sharing in organic chemistry. Routine actions that we encourage as standard practice include deposition of raw and metadata to open repositories, and inclusion of machine-readable structure identifiers for all reported compounds.

## Introduction

Fundamental to science is the ability of researchers to build upon the findings of others. Scientific data are no longer perceived as simply an output of research but as a driver for discovery. Data sharing enables researchers to find and repurpose data without the costs of repeated data collection, and secondary analysis of existing data can lead to new findings and broad impact [1–3].

Many examples of advanced practice and policy for data sharing exist in several disciplines. For example: the ‘Bermuda Principles’ in genomics [4–5], the Omics Discovery Index in biomedical science [6], access to sky survey data in astronomy [7], and commitments to data sharing in Earth and environmental sciences [8]. Sharing has led to the development of tools, standards, and data centers for these communities. The Materials Genome Initiative (MGI) [9] has accelerated the production of large, public datasets that are driving an exponential increase in the design and discovery of novel materials, their properties prediction and characterization [10–12]. Repositories for computational materials science, such as the NOvel MAterials Discovery (NOMAD) Repository [13] and Materials Cloud [14] also enable code and workflows to be stored and shared along with the data.

In the chemical sciences, sharing of thermodynamic property data is of increasing importance for process development and as a prerequisite for chemical engineering research. This is supported by the development of the ThermoML standard [15–16] together with supporting tools [17–18]. The ThermoML archive is a repository of experimental thermophysical, and thermochemical property data acquired through a collaboration with journals across several different publishers who mandate that authors share and supply their data in ThermoML format [19].

Crystallography also has an established culture of data sharing via the Cambridge Structural Database (CSD), and a research community has evolved specifically from the availability of data and the development of tools for the visualization, analysis, and exchange of crystallographic information based on a common format, the Crystallographic Information Framework (CIF) [20]. Other initiatives for utilizing shared chemistry data include the Open Science Framework (OSF) which supports the sharing of research data across disciplines and enables researchers to pre-register their studies [21], and the Open Reaction Database (ORD) which provides an open-access schema and infrastructure for structuring and sharing organic reaction data, including a centralized data repository [22–23].

With no culture of data sharing in most areas of chemistry, the reproducibility of outcomes is a recognized problem, and there are few examples of secondary analysis of open data. Common barriers to data sharing include unfamiliarity with open data concepts, poor data management training, implementation costs, lack of willingness to share, and a perceived lack of time for local database curation [24–29]. Although attitudes towards data sharing have improved over the last decade, researchers often still lack the skills, tools and incentives to share. For organic chemists, a myriad of different file formats across multiple spectroscopies are compounded by a lack of methods to collect, clean and label data in a way that makes it reusable and interoperable, especially for use by machines. Despite the barriers there are good reasons for organic chemists to share their data, to take advantage of recent advances in machine learning (ML) for synthesis planning, reaction optimization, and property prediction [30–32].

The discoverability and reusability of data, especially by machines, is central to the ‘FAIR Guiding Principles for scientific data management and stewardship’, published in 2016 [33]. These provide a measurable set of guidelines that can be used to determine the ‘FAIRness’ of shared data and are the basic framework of a developing culture of best practice. FAIR data are findable, accessible, interoperable, and reusable and this requires that raw data are deposited in open repositories, have unique identifiers, are in standard formats that are both machine-readable and easily reused, and that metadata and documentation is provided to enable others to understand who produced the data, how data were generated, and to what extent they can be reused.

Herein, we investigate the data practice of researchers publishing in specialist organic chemistry journals by analysing the data outputs of 240 randomly selected research papers from top journals. The original data that accompany the results described in these published articles are assessed according to criteria that describe if, how and where the data are shared, and whether the shared data meet FAIR guidelines.

We pose the following broad questions: Is there author compliance with recommended (but not compulsory) data policies? Do authors engage with recommendations for ‘all data’ deposition in open repositories, and are these data accessible and curated? Is there evidence to suggest that authors apply FAIR data guidance? Does the existence of specific recommendations for FAIR data practice in publishing NMR data by some journals encourage compliance? Finally, we discuss what the findings suggest about the impact of author guidelines upon researcher practice.

## Methods

12 Specialist journals with a broad scope around the central discipline of synthesis, catalysis, and methods development in organic chemistry were selected for analysis (Table 1). Criteria for journal selection are described in Supporting Information File 1.

**Table 1:** Specialist organic chemistry journals selected for this study.

Journal title	Publisher^a^

Advanced Synthesis & Catalysis	Wiley-VCH
Beilstein Journal of Organic Chemistry	Beilstein-Institut
Bioorganic Chemistry	Elsevier
Bioorganic & Medicinal Chemistry	Elsevier
European Journal of Organic Chemistry	Wiley-VCH
Journal of Organic Chemistry	ACS
Organic & Biomolecular Chemistry	RSC
Organic Chemistry Frontiers	RSC
Organic Letters	ACS
Organic Process Research & Development	ACS
Organometallics	ACS
Synthesis-Stuttgart	Thieme

^a^ACS: American Chemical Society; RSC: The Royal Society of Chemistry.

**Figure 1:**
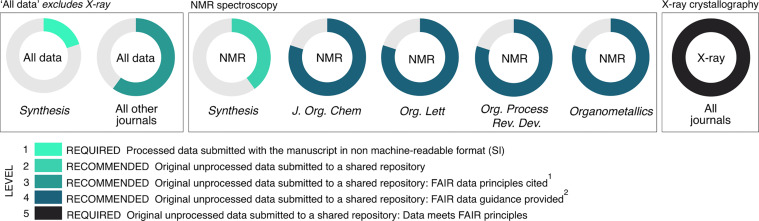
Journal data policies for the study timeframe, 01 February – 31 March 2023. ‘All data’ refers to: spectroscopic data (NMR, IR, UV–vis, Raman, circular dichroism and mass spectrometry), chromatography (GC, HPLC, SEC), physical data (m.p., b.p., elemental analysis, optical rotation), thermochemical data, computational data, structure information, cell culture and bioassay data; and excludes X-ray crystallography. ‘NMR’ refers to those examples where a distinct policy for NMR spectroscopic data is available, in addition to the ‘All data’ requirements. ^1^‘FAIR data principles cited’ indicates that FAIR data is defined and/or links to FAIRsharing resources are included in the journal guidance. ^2^‘FAIR data guidance provided’ indicates that specific requirements for open data formats and metadata/minimum information standards are included in the journal guidance.

The data policies of each of the journals were evaluated against a five–point ‘FAIRness’ scale in which the values 1–5 represent progression through the scoring levels – i.e., a score of ‘3’ indicates that the requirements of levels 1–3 have been met (Figure 1). Commonly, for any given journal, the data requirements were consistent for all data types, excepting separate requirements for crystallographic data. However, 6 journals also described separate standards for NMR data, to a higher FAIR specification than their ‘all data’ requirements.

As Figure 1 shows, whilst deposition of original unprocessed crystallography data in a repository is mandated by all journals, submission of unprocessed NMR and other data are only recommended, with varying degrees of guidance on FAIR practice. Journal data policies for publication of crystallographic data are excluded from the ‘all data’ group which combines the other categories, because an excellent data culture is already established for crystallographic data publishing. All journals expect CIF validation using accessible tools (checkCIF [34] or enCIFer [35]), and deposition of CIF files and structure factor tables with the Cambridge Crystallographic Data Centre (CCDC), enabling full access to the contained structure information via the CSD repository. For other data types, 11 of the 12 journal titles recommend that primary data are deposited in a repository and encourage authors to consider FAIR data practices. It is nonetheless the case that the compulsory requirements stipulate only that authors supply processed data in a non-machine-actionable format (usually a supporting information PDF file), alongside descriptions of post hoc analyses within this same format.

Of the five journals that make a further recommendation for repository deposition of primary NMR data at the authors’ own discretion, the four American Chemical Society (ACS) titles provide a description of FAIR file formats and the minimum information that constitutes well–structured raw data (free induction decay (FID) files, acquisition and processing parameters), and metadata (spectrometer specifications, acquisition and processing software, and sample information). Two of the ACS journals, *J. Org. Chem.* and *Organometallics*, give instructions for preparing data files of Cartesian coordinates from computational studies, requiring that these are provided in XYZ and MOL formats.

Although the author guidelines often encourage repository deposition of data (9 journals recommend the use of subject-specific repositories), little guidance is provided other than for crystallographic data. None of the journals suggested a subject-specific repository for NMR data, and an update to the RSC data sharing policy in April 2024 after the sampling window, recommends the use of only a generic or institutional repository for FID data. This is consistent with the findings of Parks et al. who examined author guidelines from 42 journals and found that while 68% recommend storing data in a subject-specific repository, only 32% indicate specific NMR repositories [36].

Sampling of journal articles in the two-month window of 01 Feb – 31 Mar 2023 was carried out as follows:

A glossary of ‘article types’ defined by each journal as constituting original research was compiled and, for each journal, a vector of integers was generated in RStudio [37] corresponding to the total number of original research articles published in 2 months (excluding review articles). From the vector for each journal, random sampling was used to select 20 articles, i.e., by selection of 20 random integers and matching of these to a chronological list of the articles. Assessment of the data objects associated with the selected articles was then carried out. The inclusion of 18 possible data types generated in each research article was recorded using Yes/No binary responses. Each research article was then assessed against 9 categorical variables that describe the main features of the paper and its associated data, and against 17 ‘FAIR’ variables that measure the extent to which the data meet FAIR data standards [38].

These FAIR variables are defined in Table 2. The coding of responses, list of data types associated with each article, and the resulting main dataset from assessment of 240 research papers are available in our supporting data package. As all research articles include results based on original (raw) data, and include previously unreported chemical structures, every article was assigned a response to the criteria defined in Find_1, Find_4, and Access_3. Then, all remaining variables in Table 2 were assessed only for those studies where primary data had been shared, as established by Access_3.

**Table 2:** FAIR variables assessed in the analysis.^a^

	Variable	Description

Findability	Find_1	The dataset is deposited in an open repository
	Find_2	The associated article is cited or linked
	Find_3	The dataset is assigned a unique, citable and persistent identifier (i.e., DOI)
	Find_4	All compounds are assigned a unique, citable and persistent identifier (i.e., SMILES or InChI + InChI key)
	Find_5	Data-time stamps for creation, deposition and versions of the dataset are included
	Find_6	Bibliographic metadata are available (authors, contributors, affiliations, funding source)
Accessibility	Access_1	Coherent file structure (logically grouped and labelled files)
	Access_2	README file describes the dataset
	Access_3	Unprocessed primary data are included
	Access_4	Metadata describe instrument parameters (vendor, model, version, software)
	Access_5	Metadata describe experiment parameters
	Access_6	Metadata describe data cleaning/analysis/processing/visualisation method(s)
Interoperability	Inter_1	Original data are in standard open format(s)
	Inter_2	Metadata are in a machine-readable format (i.e., XML, JSON)
Reusability	Reuse_1	Original data are in reusable format
	Reuse_2	Open licence information is available (i.e., CC or ODC) under which data can be reused
	Reuse_3	Code scripts necessary to reproduce findings are available

^a^All responses are Yes or No, except for Access_3 where the response is ‘NONE’, ‘COMPLETE’ for all data reported in the main paper, or ‘PARTIAL’ for selected experiments.

## Results

### Data types

**Figure 2:**
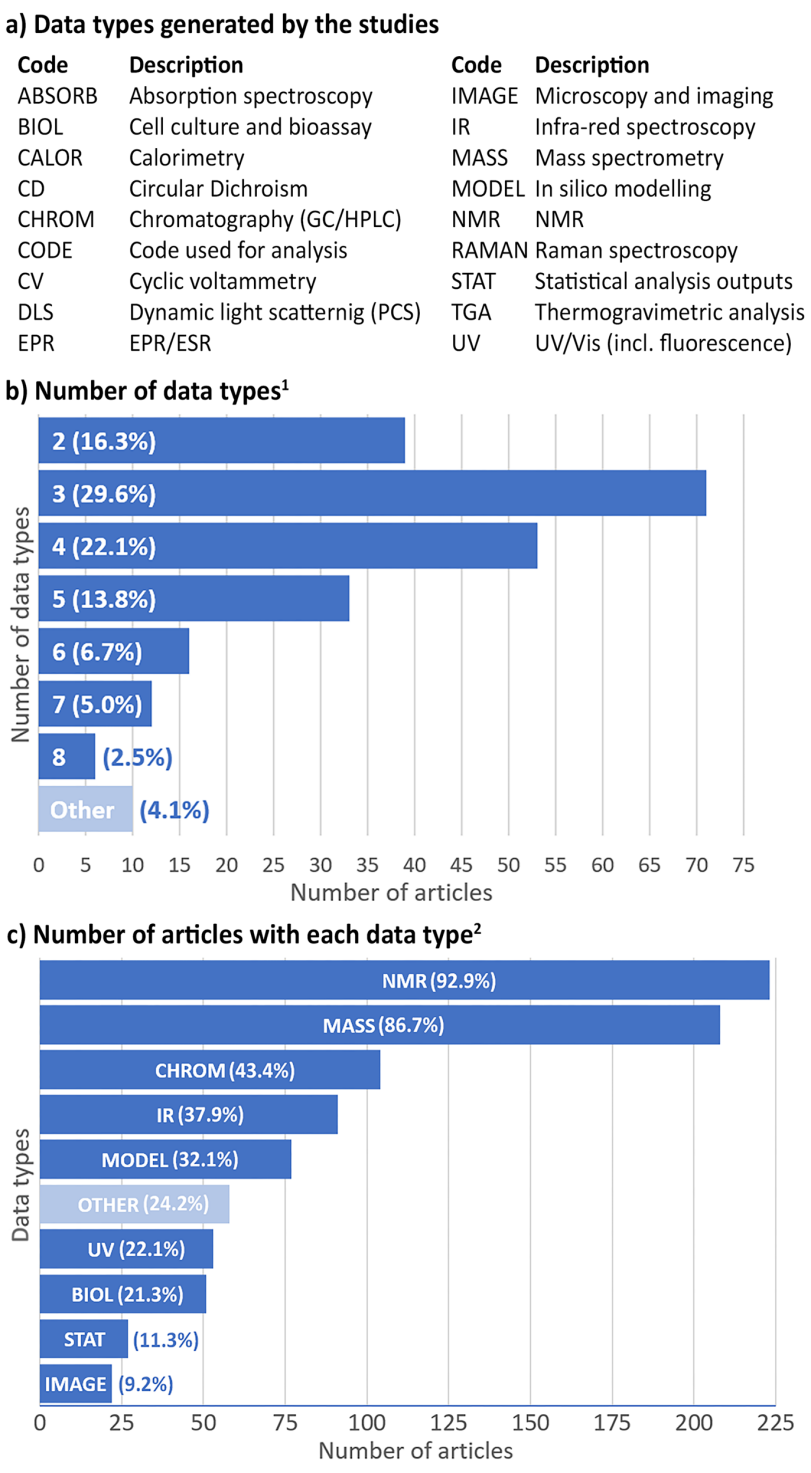
Data types and counts. ^1^’Other’ combines articles with 0 (0.4%), 1 (3.3%) or 10 (0.4%) types of data. ^2^‘OTHER’ combines articles with ABSORB (2.1%), EPR (2.1%), RAMAN (0.8%), CD (4.2%), CALOR (5.0%), CV (6.7%), TGA (2.1%), DLS (0.4%) and CODE (0.8%). Data codes are defined in panel (a). Those articles not reporting NMR (7.1%) or MS (13.3%) are those for which these data were not relevant to the work.

More than 95% of research articles report at least two types of data (Figure 2a,b). Commonly, studies generated NMR data (93%) and mass spectrometry (MS) data (87%) as these are the minimum requirement for compound characterization in all journals (Figure 2c).

### Are unprocessed primary data included? (Access_3)

**Figure 3:**
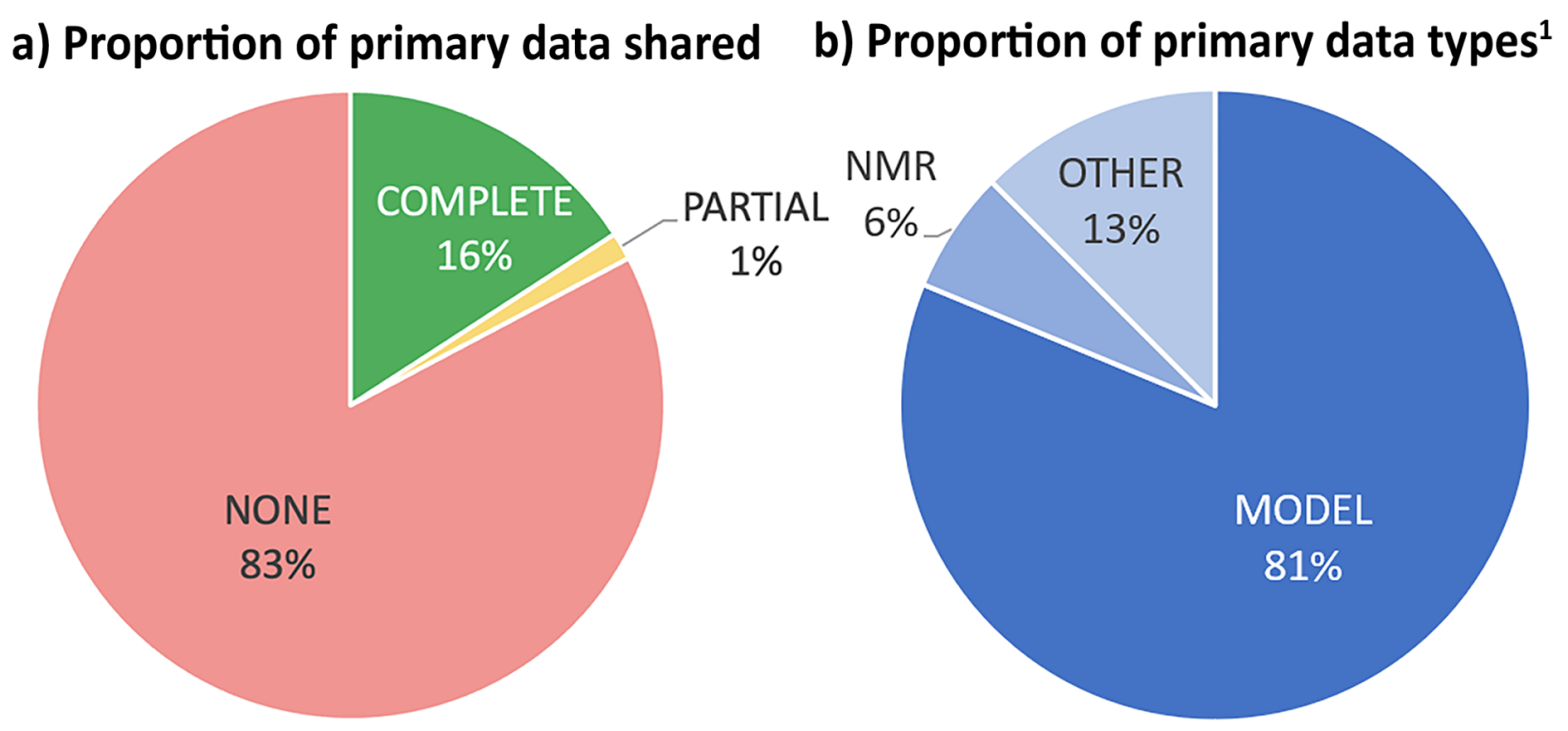
Primary data sharing. ^1^Only two papers shared >1 type of primary data. ‘OTHER’ combines articles sharing (one of) STAT (2%), BIOL (4%), CODE (2%) or (IMAGE (4%) data types. Data codes are defined in Figure 2(a).

239 of the 240 articles produced data, but only 47 shared any primary data at all (Figure 3a). Of these, most (39) shared ‘MODEL’ data derived from in silico modelling – i.e., Cartesian coordinates associated with modelled structures in thermochemical calculations or binding studies (Figure 3b).

**Figure 4:**
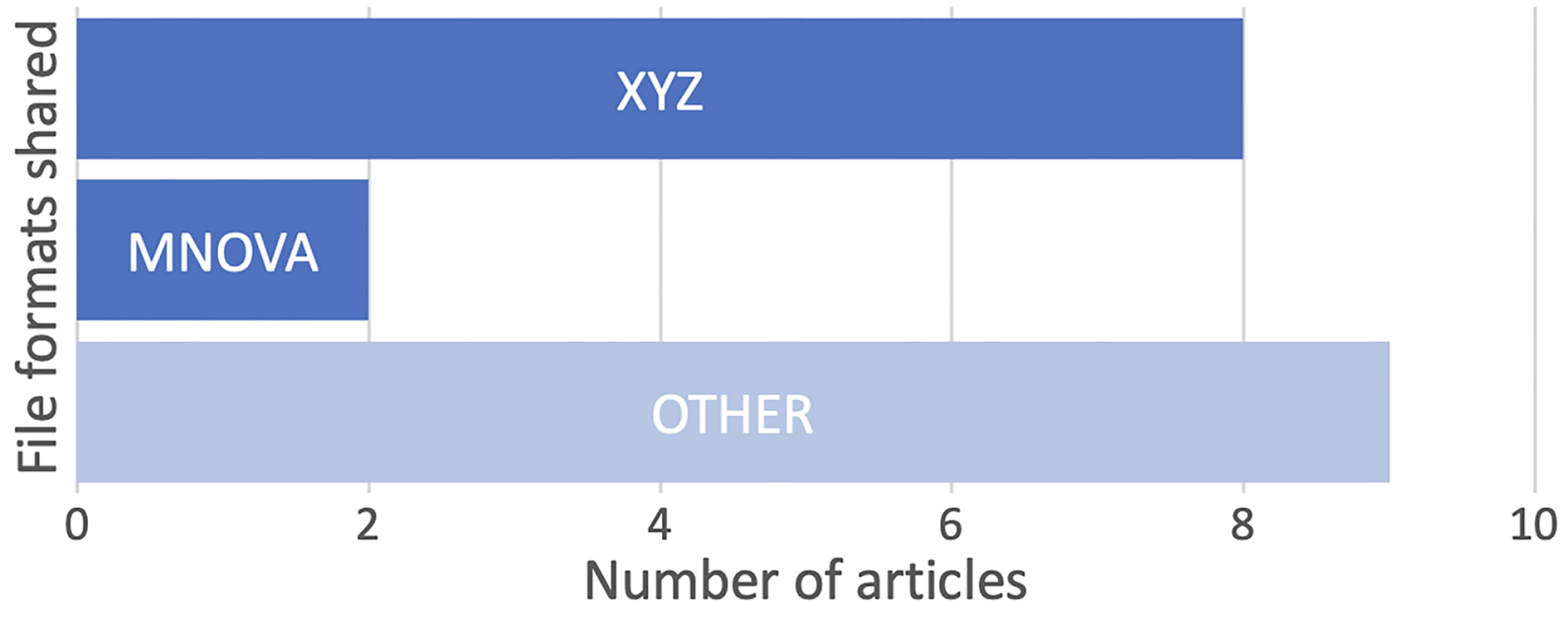
File formats supplied as SI (excluding PDF and Word DOC). ‘OTHER’ combines articles sharing (one of) CSV, GROMACS, IPYNB, JDX, JPG, TXT, MAE, SCN and XLSX.

72% of these coordinates were given in PDF format, in SI files, and only 21% in standard XYZ format. Overall, 63% of the shared primary data were included in an SI file rather than in a data file. For data types that were provided in non-PDF file formats, XYZ coordinates files are the most common, and all were published in *Organometallics* and *Eur. J. Org. Chem*, where XYZ files for computationally derived structures are mandated (Figure 4). Of the 223 research papers to report NMR data, only 3 shared primary data (as MNOVA or JDX files) with none providing raw FID data despite encouragement from the journal guidelines. Note that ‘JDX’ is a file extension for JCAMP-DX, the IUPAC standard file format for spectral data [39]. FIDs are accessible from the MNOVA and JDX files, but access requires additional software. No primary mass spectrometry data were shared.

### Are data deposited in an open repository? (Find_1)

**Figure 5:**
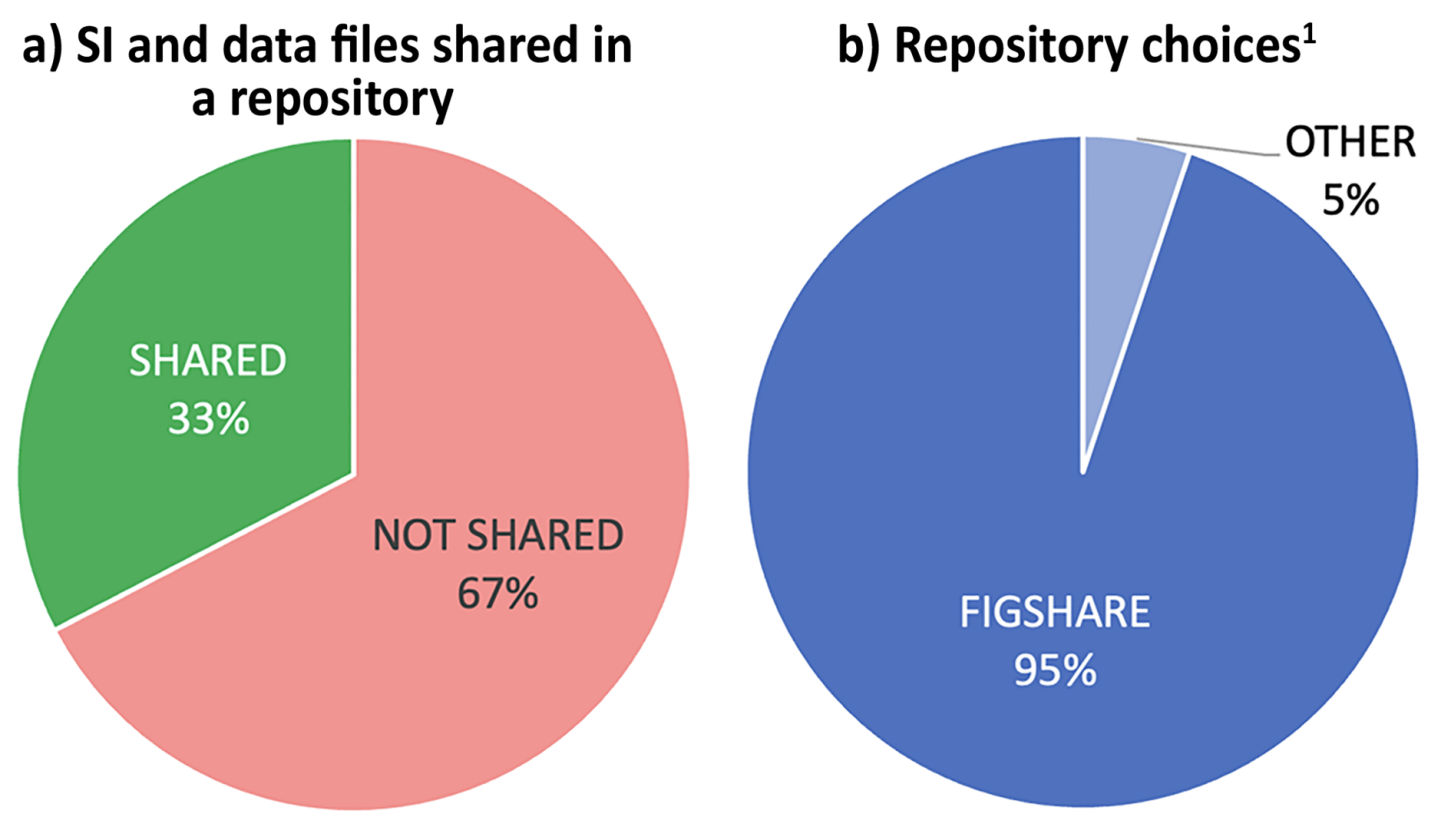
Repository deposition. ^1^’OTHER’ combines Github (1.3%), institutional repositories (1.3%) and Zenodo (2.6%).

Two thirds of shared primary data were not deposited in a public repository (Figure 5a). Except for one example from *Synthesis*, data of any type had only been shared to a public repository when published in one of the four ACS journals. 95% of these used Figshare [40], (Figure 5b), and this is entirely publisher-led behavior as all ACS journals automatically assign a DOI and upload SI files to Figshare. Although 75% of journal guidelines recommended that data should be deposited in a subject-specific repository, the authors of only 4 articles (<2%) shared data in a public repository, and none used a subject-specific repository that would provide specialist functionality, such as structure searching, to facilitate discovery for other chemists.

We conclude that recommending (not mandating) repository deposition does not encourage data sharing. Interestingly, *Eur. J. Org. Chem.* 'expects’ that the data supporting the reported results are archived in a public repository and the scripts and other artefacts used to generate the analyses presented in the paper ‘should’ also be publicly archived. These are strong terms that suggest the journal requires data to be deposited, but no author of an article included in our study had done so.

### Are all compounds assigned a unique, citable and persistent identifier? (Find_4)

None of the 240 articles examined, or their associated SI files, included a unique, citable, and persistent identifier for any compound, even though Simplified Molecular Input Line Entry System (SMILES), International Chemical Identifiers (InChIs) and InChIKeys are widely supported in software handling chemical information such as databases, inventories, laboratory information management systems (LIMS) and electronic lab notebooks (ELNs). Systematic naming of organic compounds using International Union of Pure and Applied Chemistry (IUPAC) nomenclature was widely used. Although syntax analysis could be considered an option for parsing these names, this would rely on their provision in a machine-readable text format and compound naming was provided in the PDF format of the supporting files for all articles. The lack of structure identifiers highlights the culture change that is required to reverse the loss of machine-readable compound identifiers when data are prepared for publication.

#### Adherence to FAIR principles

**Figure 6:**
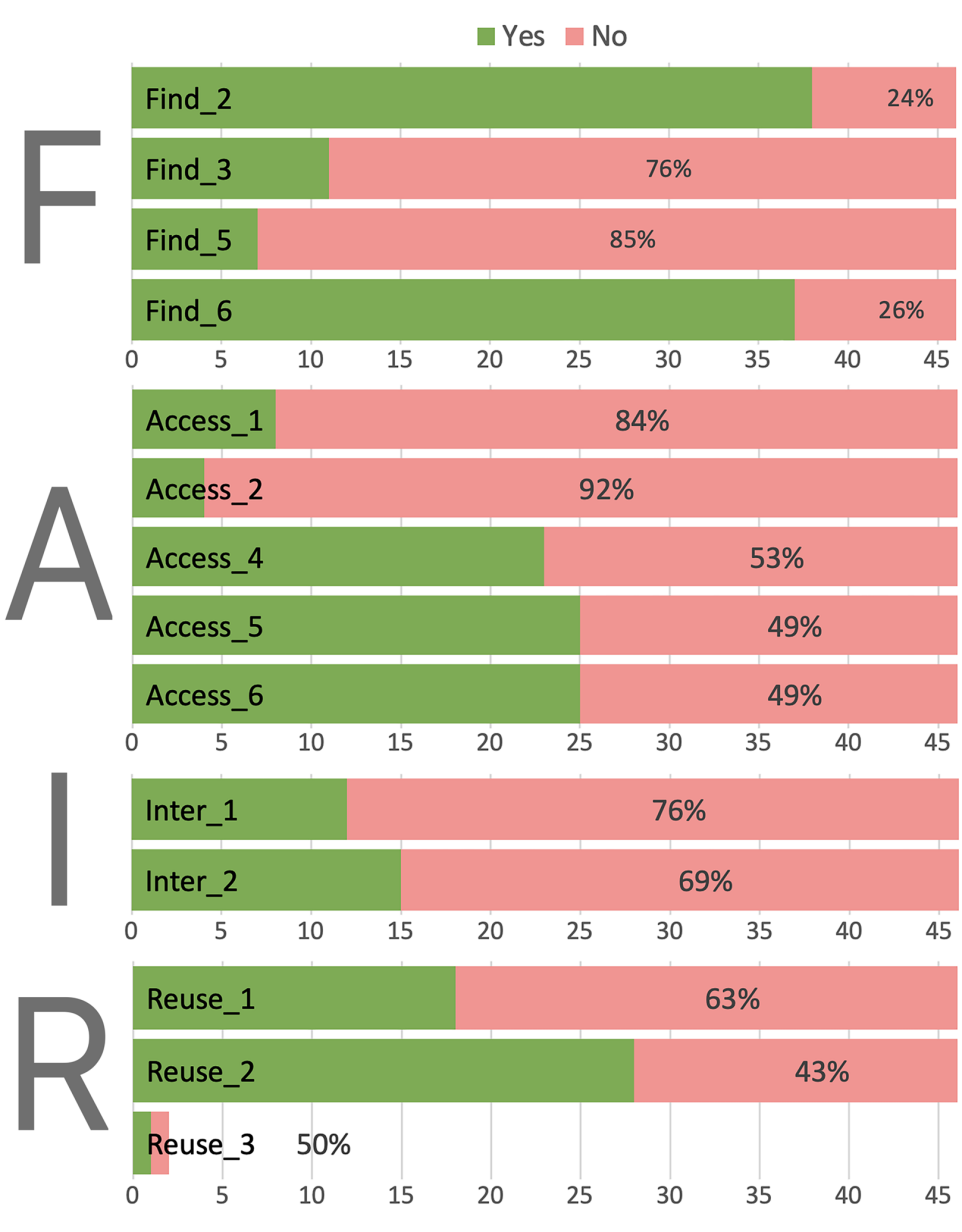
Compliance of shared data with FAIR principles. FAIR variable codes with the form ‘Name_integer’ are defined in Table 2.

For the 47 research studies that shared primary data, these outputs were assessed against the remaining ‘FAIR’ variables given in Table 2, to measure the extent to which the data meet FAIR data standards. The results are summarized in Figure 6, and in the following results subsections: Findability, Accessibility, Interoperability and Reusability.

#### Findability

Find_2, ‘*is the associated article cited or linked?*’ assesses if it is possible to trace data back to the original publication where the methods used to generate those data are described. 76% of primary data had a citation, however, publisher-led copying of SI files to Figshare positively distorts this proportion, as the repository landing page provides this link back to the main article.

Find_3, ‘*is the dataset assigned a unique, citable and persistent identifier?*’ assesses if the data can be found over the long-term. We found poor compliance (24% of primary data) as only files uploaded to a formal repository had a DOI. Two datasets uploaded to Zenodo [41], one data package deposited with an institutional repository, and MODEL data uploaded in native formats had a unique identifier assigned by the repository for the data or dataset. None of the SI PDFs containing MODEL data uploaded to Figshare can be considered to have a DOI specific to the primary data.

Find_5, ‘*are Date-time stamps for creation, deposition and versions of the dataset are included?*’ None of the articles sharing primary data had timestamps for all these elements.

Find_6 ‘*are bibliographic metadata available?*’ relates closely to Find_2 and as a result mirrors our findings of whether there is a link to the original article, which includes author affiliation and bibliographic data.

#### Accessibility

Access_1, ‘*is there a coherent file structure*’ assesses whether primary data shared in native formats are appropriately grouped and named. Sensible file naming occurred for 6 articles, only when the data had been packaged by authors or placed on a repository where the authors were able to name the files and select an appropriate directory structure. So, with autonomy over curation of a mandated data package, sensible naming and file structures is made.

Access_2, *‘does a README file describe the dataset?’*. The primary data of only two articles was accompanied by a README, both were for data stored in a repository. The README is an important file that provides essential information for users of shared data such as provenance details, instructions on how to use the data, and license information. Guidance on how to create and structure a README file is provided in the Supplementary Information for this article.

Access_4, ‘*do metadata describe instrument parameters (vendor, model, version, software)?*’ and Access_5, ‘*do metadata describe experiment parameters?’* assess if there are enough metadata to understand how the data have been produced and whether they have been subject to any modification. Around half of the primary data files gave metadata describing instrument and software specifications, and we note that instrument and experimental parameters are intrinsic to NMR data in JDX format. MNOVA gives some of these metadata in the file headers, and SCN, MAE, or MDP image files also report some parameters. However, proprietary formats do not enable full, open access to all the relevant experimental, instrument and software information.

Access_6, ‘*Do metadata describe data cleaning, analysis, processing and visualization?*’. Although some of the necessary information was given in 22 SI PDFs reporting primary data, it is difficult to extract and not linked directly to the data. The metadata were not found within the native data files, and therefore if the primary data were separated from the original article or accompanying SI file that context would be lost. An exception is the one article that had primary data along with code deposited in GitHub [42], where the Jupyter Notebook file [43] provides the code used to process the data and therefore documents the operations.

#### Interoperability

Interoperability considers how easy it is to take the data and integrate with other data, or reuse in existing applications and workflows.

Inter_1 ‘*are original data in standard open formats?*’. Just 16 articles (7%) shared primary data in native formats, of which 10 used open formats, PYNB [42], JDX [44], CSV, XYZ and TXT. The Jupyter notebook file PYNB is a standard open file type in JSON format, so the code can easily be read by machines. JDX, the JCAMP-DX standard championed by IUPAC [39], is the only community accepted standard of these open formats, and the three other file formats have challenges for interoperability. The most common native file format to be shared was coordinate XYZ data. This file is human and machine readable, and although well supported by software in the chemical sciences, a specification has not been published such that several different formats exist. CSV also has no formal specification, and different characters and delimiters are used by different applications.

Inter_2 ‘*are metadata in a machine-readable format?*’. Less than a third of shared primary data files have machine-readable metadata content. In addition to the open formats JDX, XYZ, CSV and PYNB, the image files SCN, MDP and MAE provide metadata in XML or text format as part of the file headers.

#### Reusability

Reuse_1, ‘*are original data in a reusable format?*’. The primary data shared in native file formats by the authors of only 16 articles were in a reusable proprietary format, although requiring access to the original software application or to conversion tools, such as Open Babel [45].

Reuse_2, ‘*is open license information available?*’ checks the availability of a license detailing permission for reuse of data, and any conditions associated with that reuse. 27 publications had some license information, but this was associated with primary data in only 3 articles. These were publisher-led, with MODEL (coordinates) data shared in SI PDF files associated with articles in *Beilstein J. Org. Chem.,* where both article and SI files include a ‘boilerplate’ license that describes the Beilstein-Institut Open Access License Agreement. *Eur. J. Org. Chem.* encourages the use of open licenses and has a boilerplate license statement on the first page of an article under the terms of the Creative Commons Attribution Non-Commercial License [46], although this license is not replicated for the SI files, leaving ambiguity as to the status of the reuse of data enclosed therein.

Files uploaded to Zenodo [40] can associate a license with the landing page for the data in the repository. Of the two data sets uploaded to Zenodo, authors of one article had chosen to set a license and one had not. The data uploaded to an institutional repository do include an overarching license on the repository website. The data and code uploaded to GitHub [41] do not have any associated license information. Of the data files or packages that were not uploaded to a repository, none included a license. The SI PDFs and native data files uploaded to Figshare [39] from the ACS journals have CC0 [47] assigned as the default license, meaning that the data have been dedicated to the public domain and others can reuse the data in any way they want to without citation of the source. It is unclear whether authors can select from alternative licenses or create a tailored license for their data.

Reuse_3, ‘*are code scripts necessary to reproduce findings available?*’. For data that had been generated through software processing, algorithms or other calculations performed on primary data, only one paper shared any code. Statistical outcomes shared in one Excel file reported only values, with no calculations.

## Discussion

We frame our discussion (below) according to the four research questions posed in our introduction to this work and provide specific recommendations for strategies towards improved standards in data publishing therein. We also comment upon action researchers can take to move towards a positive culture of data sharing in organic chemistry, and signpost relevant resources.

### Is there author compliance with recommended (but not compulsory) data policies?

**Figure 7:**
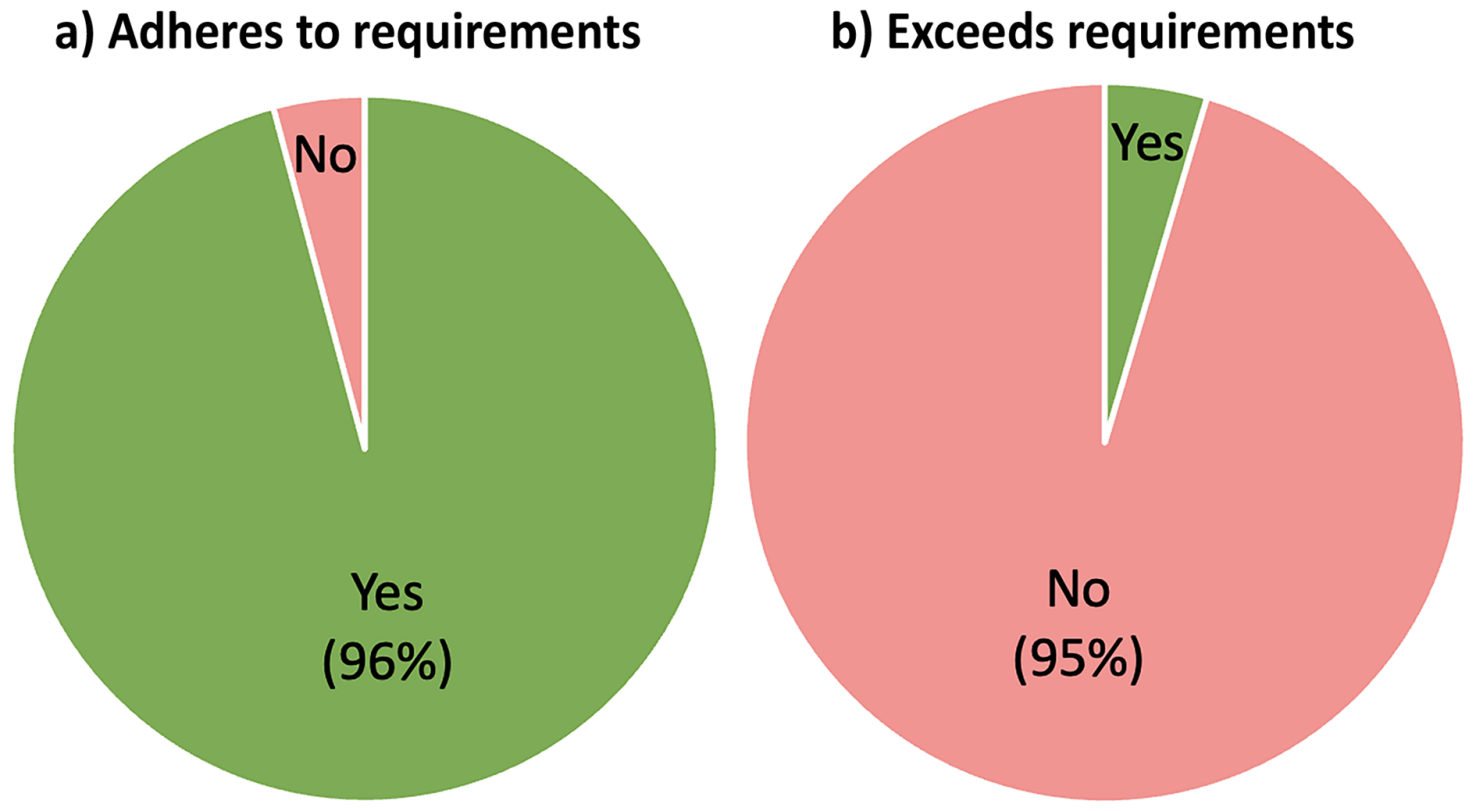
Proportions of articles that a) meet and b) exceed, publisher data requirements.

Compliance with mandatory publisher requirements is excellent (96%, Figure 7). However, we view this in the context that low ‘FAIR’ data standards are mandated by journals. Compliance with policies that merely recommend a higher standard of data sharing is very poor, authors of only 8 articles shared primary data that was not mandated. Excluding crystallographic data, authors of only 45 articles, less than 20% of those analyzed, shared any primary data at all, and of those only 16 articles had shared primary data in a native format outside of a PDF file. The authors of just one article shared any kind of code that would enable another researcher to verify the research outcomes or apply methods to their own data. Our results suggest that journals have a clear role to play in promoting data sharing and FAIR practice. Ideally, compulsory data sharing policies will include guidance on the use of repositories and file formats to agreed common standards. Mandating specific repositories and file formats will increase the likelihood of adoption, and of funding for continued repository curation, but these choices of repositories and data formats require community consensus. For individual journals, there is a clear risk that enforcing data requirements without community standards will go too far, too quickly. So, to avoid a decrease in submissions by researchers in response to the burden of ‘FAIRification’, publisher consensus on data sharing policies is also required.

Despite the challenges, we have shown a clear need for improvement to the mandated data sharing journal guidelines, that could be achieved via a common data policy framework for all publishers [48].

### Do authors engage with recommendations for ‘all data’ deposition in open repositories and are these data accessible and curated?

Engagement with journal recommendations for repository deposition of data was overwhelmingly poor. Excluding the publisher-led deposition of SI files to Figshare [39] associated with ACS journals, the authors of only 4 articles (<2%) shared data in a public repository – of which none are subject specific. Reasonable curation and accessibility of these datasets is in place.

Of note, is the distinction between best practice in data sharing of crystallographic cf. other data types in organic chemistry. An established culture of data sharing in crystallography has developed from establishment of a centralized repository and the CIF common standard, adopted in 1990 [49]. Requirements for the production and sharing of crystallography data are clear and consistent, and researchers are often supported by staff in crystallography services, where the data are curated and provided to researchers in appropriate formats for sharing, and in many cases the crystallographers also do the work of obtaining a checkCIF report and submitting the data to CCDC. The researcher need only supply Accession numbers on publication. For other data types, researchers are on their own. We return to our earlier point that, following the example of crystallography, a ‘FAIR sharing’ culture for other data types requires standard formats and centralized repositories. Support for researchers to reduce the burden of curation would encourage best practice, and we foresee a change in the culture and funding of analytical services, to involve data curation by NMR and mass spectrometry services that are typically staffed and embedded as distinct units within chemistry departments, to mirror the practice of crystallography services.

### Is there evidence to suggest that authors apply FAIR data guidance in their data publishing practice?

There is little evidence of authors applying FAIR data guidance in their data publishing. Current practice involves minimal primary data sharing and predominantly ties up those data that are provided in PDF files. Of four articles where authors had intentionally uploaded their data to a repository, none showed a strong understanding of how to prepare the data to maximize its interoperability and reusability. In some areas, practice ‘met’ FAIR guidance only superficially. For example, for the two articles that provided READMEs, the content lacks useful metadata and documentation. The authors of these articles used GitHub, which strongly encourages the creation of a README via the interface once a repository has been created, and in the documentation. The GitHub README is often the first item that a visitor to the repository will see, and therefore GitHub recommends that repository owners include useful descriptive information of their project, although the guidance is very limited. The prominent prompt to create the README and the associated lack of detailed advice on what to include could explain why a README exists, but also why it is insufficient to adequately describe the data contained therein.

Only one article shared primary data in a format recognized as a formal standard in chemistry, and although others had some elements of metadata that could be extracted, the proprietary nature of the other formats mean they are not interoperable and reusable. Only two primary data packages contained information to direct a user to the original article once those data had been downloaded; and none had an associated data DOI or link to the article DOI. Metadata, typically embedded in SI associated with the article on a publisher site, are lost.

A striking finding of our work is that none of the articles contained structure identifiers for their compounds. Most journals include guidance on nomenclature, and commonly recommend systematic naming based on Chemical Abstracts Service (CAS) and IUPAC nomenclature. But the use of general descriptors such as ‘polypeptide 14a’ is common practice, culturally embedded for organic chemists and important for clarity and readability of the article. Unfortunately, ‘trivial’ compound naming is also widely accepted for naming across supporting files, and several journals actively discourage the use of computer-generated multi-line or systematic compound names. While the intention is to preserve clarity and uniformity across the associated files, this practice concurrently discourages the use of InChI [50] and SMILES [51] identifiers. A lack of unambiguous structure identifiers makes it difficult (or impossible) to extract the associated spectroscopic data or properties for reuse through computational means. Overall, the lack of machine-readable compound names across all journals suggest that the researchers and publisher communities do not currently prioritize data sharing requirements for machine discoverability and reusability. We encourage new policies to incorporate compound identifiers (e.g., SMILES, InChIs and InChIKeys) alongside trivial names so that both humans and machines can read the data.

### Does the existence of specific recommendations for FAIR data practice in the publishing of NMR data by some journals encourage compliance?

The widespread practice of sharing NMR data only in processed form denies the research community the opportunity to re-examine the data or subject it to analysis using new techniques. FID is the recommended format to share primary data, cited by the ACS journals and *Synthesis*. Of the 223 studies included here that produced NMR data, only 3 shared primary NMR data: 1 each in an ACS journal, *Synthesis*, and an RSC journal. The acquisition and processing parameters were not included and none of these data included FID files. So, the provision of NMR sharing recommendations (and including detailed guidelines from ACS) does not lead to author compliance in the studies we have examined. Various open repositories for NMR data exist, including nmrXiv [52], NP-MRD [53], BMRB [54], and nmrshiftdb2 [55], but no repository has established widespread use to date. Most instrument vendors output FID data in proprietary data formats which can be opened by most analysis software, but many also export the data in (open) JDX format which has a specification for FID raw data. Opportunities to progress the FAIRification of NMR data sharing are therefore in place, and we further discuss these opportunities from the researcher standpoint, below.

#### Cultural change

Cultural change will follow from a community of researchers that have evolved to acknowledge the benefits for funding and discovery that are enabled by FAIR data [56–57]. Structured support, infrastructure and policy will enable this process, and many initiatives are underway. Standardization for file formats and good practice under development by IUPAC include InChI (structure-based chemical identifier) [50], ThermoML (standard for thermodynamic property data) [16], and JDX (spectra exchange information) [44] for a wide range of analytical data, and the specification for FAIR management of spectroscopic data [58]. Other organizations working on standards include NFDI4Chem [59] (analytical data standards), and Pistoia Alliance (Unified Data Model, or UDM, for compound synthesis and testing) [60]. GO FAIR [61], CODATA [62], and the Research Data Alliance [63] promote data sharing best practice and RO-Crate provides guidance on how to package and store data [64].

As the required supporting resources develop, policies for mandatory data sharing to FAIR standards will soon follow, from institutions and funders. It will be vital that these policies are supported by appropriate recognition and incentives that reward researchers for their time spent in adapting to the coming, higher, standards for data curation. Credit through data citation will play a role that replicates the existing recognition of researchers by their publication citations, and institutions should expect to be measured on how well their researchers are supported.

At present, as rigorous data-sharing policies imposed by publishers, institutions and funders are in a development stage, any individual researcher is hardly expected to explore disparate and multiple FAIR data projects – of which just a few are cited above. In the data package associated with this paper [65], we provide links to minimum information checklists, recommendations, and standards (including open file formats and repositories) for the main data types relevant to organic chemists (see: Metadata_checklist_resources.doc). Below, we make some general recommendations of first steps and key resources:

#### Actions researchers can take

*Plan for data sharing.* It is time consuming to prepare data for sharing retrospectively, a Data Management Plan (DMP) encourages researchers to consider data management in advance of data collection. DMPOnline [66] provides a wizard tool to create a DMP, along with resources such as public DMPs and information about funder requirements.

*Share primary data in a public repository*. Use a repository that provides a persistent identifier (e.g., DOI) so that the data can be cited, by you in the main article and by others that reuse the data. Before depositing or reusing data, consider how well the repository meets the Trust Principles: Transparency for best practice in digital preservation [67]. Many generic repositories accept research data for free, including Figshare [39], Zenodo [40], Open Science Framework [68], Dryad [69], and Mendeley Data [70]. There are several specific repositories for NMR data including: nmrXiv [52], BMRB [54], and nmrshiftdb2 [55]; and the RSC now recommends deposition of a zip file of raw instrument data (the entire file directory for the experiment, including the FID and associated files), with processed spectra optionally included. MassBank is a specific repository for small molecule and metabolomics mass spectral data [71–72].

*Use open file formats*. Where possible, do not provide data in proprietary formats. Become familiar with the data formats and standards in your area of research, IUPAC provide information about many standard formats [73]. Open formats in NMR, mass spectrometry, UV–vis-, IR, and Raman spectroscopy have been recently reviewed [74]. Often a very simple first step is to export coordinate spectroscopic data to CSV, and there are numerous online tools for conversion of proprietary to open file formats – for example Open Babel [45], which provides tools for interconversion of >100 file formats common in the chemical sciences.

*Document and share metadata.* During the data collection stage, prepare the metadata that describe bibliographic information (contributors, ORCiD iDs, affiliations, funding sources); date and timestamps; instrument, software, processing and experimental parameters; and data dictionaries for primary data or code where variable names require delineation. A machine-readable README file should enable others to view and reuse the data, including license information.

*Include structure identifiers.* In addition to the trivial naming of compounds that is necessary for a human reader to clearly understand the work, also provide linked non-proprietary and machine-readable identifiers (e.g., SMILES or InChIs and InChIKeys) [50–51] to facilitate machine discoverability and reusability.

## Conclusion

An examination of the data sharing practices of authors across 240 organic chemistry papers indicates that less than 20% of articles have any associated primary data, and these are predominantly Cartesian coordinates associated with in silico modelled structures, generally shared in non-machine-readable PDF format. Only 1% of studies shared unprocessed NMR data; despite >93% of research articles reporting NMR data as a main output of their work, and the existence of strongly worded recommendations for NMR data-sharing in the author guidelines of 5/12 journals.

We find that, for the overwhelming majority of articles, only mandatory data requirements are met. This suggests that the positive promotion of FAIR principles by the cheminformatics community has had little effect amongst mainstream researchers to date, and journals/publishers have a key role to play in driving an improved data-sharing practice amongst researchers in organic chemistry. At the time of publication of this work, publishers’ data-sharing policies continue to present researchers with the same optional (not mandated) requirements for FAIR-sharing that were summarized in Figure 1 at the commencement of our study. We note very few updates to author guidelines since then: ACS now states that submission of primary NMR data files is “highly recommended” to potential authors of publications in *Org. Lett.* and *Organometallics*, and is “requested” for publication in *J. Org, Chem.*; while the RSC now “encourages” authors to submit a summary of compounds reported in any submitted manuscript that should include SMILES, InChI and InChIKey identifiers. Lastly, Beilstein-Institut has updated author guidance with signposting to registries of subject-specific open repositories.

These changes are modest in the least, and significant culture change is required to develop author familiarity with FAIR data principles and promote their application. This cultural change must be supported by mandated sharing of key data types, promotion of common file formats, support for researchers to reduce the burden of curation, and incentives for researchers to report the outcomes of low/no-yielding reactions that describe a broad chemical space [31,75]. Mandates for deposition, standardized data formats, and centralized repositories are all necessary to enable compliance with FAIR principles [76]. AI is having a major impact on many aspects of the world we live in but will have little (or no) impact in the chemical sciences as long as humans (let alone machines) do not have access to properly contextualized and trustworthy data. Herein, we have evidenced that current practice does not support machine-augmented discovery. Our community risks becoming left behind without urgent cultural change.

## Supporting Information

File 1 Criteria for journal and article selection, R code for journal sampling, article assessment criteria, files included in the supporting data package, advice for creating a README file.

## Data Availability

Data generated and analyzed during this study are openly available in Zenodo at: https://zenodo.org/records/13928084 (directs to the version at time of submission). Cite: Bloodworth, S.; Willoughby, C. & Coles, S. J. (2024). Data accessibility in the chemical sciences: an analysis of recent practice in organic chemistry journals (2.0) [Data set]. Zenodo. https://doi.org/10.5281/zenodo.11068278. The data package contents are described in the associated README file and the Supporting Information PDF. The data is licensed under CC BY 4.0.

## References

[1] Bonàs-Guarch S, Guindo-Martínez M, Miguel-Escalada I, Grarup N, Sebastian D, Rodriguez-Fos E, Sánchez F, Planas-Fèlix M, Cortes-Sánchez P, González S (2018). Nat Commun.

[2] Piwowar H A, Day R S, Fridsma D B (2007). PLoS One.

[3] Milham M P, Craddock R C, Son J J, Fleischmann M, Clucas J, Xu H, Koo B, Krishnakumar A, Biswal B B, Castellanos F X (2018). Nat Commun.

[4] Maxson Jones K, Ankeny R A, Cook-Deegan R (2018). J Hist Biol.

[5] Kaye J, Heeney C, Hawkins N, de Vries J, Boddington P (2009). Nat Rev Genet.

[6] Perez-Riverol Y, Zorin A, Dass G, Vu M-T, Xu P, Glont M, Vizcaíno J A, Jarnuczak A F, Petryszak R, Ping P (2019). Nat Commun.

[7] Pepe A, Goodman A, Muench A, Crosas M, Erdmann C (2014). PLoS One.

[8] Michener W K (2015). Ecol Inform.

[9] (2025). Materials Genome Initiative.

[10] de Pablo J J, Jackson N E, Webb M A, Chen L-Q, Moore J E, Morgan D, Jacobs R, Pollock T, Schlom D G, Toberer E S (2019). npj Comput Mater.

[11] Morgan D, Jacobs R (2020). Annu Rev Mater Res.

[12] Choudhary K, DeCost B, Chen C, Jain A, Tavazza F, Cohn R, Park C W, Choudhary A, Agrawal A, Billinge S J L (2022). npj Comput Mater.

[13] (2025). NOMAD.

[14] (2025). Materials Cloud.

[15] Frenkel M, Chiroco R D, Diky V, Dong Q, Marsh K N, Dymond J H, Wakeham W A, Stein S E, Königsberger E, Goodwin A R H (2006). Pure Appl Chem.

[16] (2025). ThermoML.

[17] (2025). ThermoData Engine.

[18] (2025). ThermoPlan.

[19] (2025). ThermoML Archive.

[20] (2025). Crystallographic Information Framework.

[21] (2025). Open Science Framework.

[22] Kearnes S M, Maser M R, Wleklinski M, Kast A, Doyle A G, Dreher S D, Hawkins J M, Jensen K F, Coley C W (2021). J Am Chem Soc.

[23] (2025). ORD, Open Reaction Database.

[24] Tenopir C, Rice N M, Allard S, Baird L, Borycz J, Christian L, Grant B, Olendorf R, Sandusky R J (2020). PLoS One.

[25] Chawinga W D, Zinn S (2019). Lib Inf Sci Res.

[26] Tenopir C, Christian L, Allard S, Borycz J (2018). Earth Space Sci.

[27] Alharbi E, Skeva R, Juty N, Jay C, Goble C (2021). Data Intell.

[28] Borycz J, Olendorf R, Specht A, Grant B, Crowston K, Tenopir C, Allard S, Rice N M, Hu R, Sandusky R J (2023). Humanit Soc Sci Commun.

[29] Perrier L, Blondal E, MacDonald H (2020). PLoS One.

[30] Davies I W (2019). Nature.

[31] Strieth-Kalthoff F, Sandfort F, Segler M H S, Glorius F (2020). Chem Soc Rev.

[32] Tu Z, Stuyver T, Coley C W (2023). Chem Sci.

[33] Wilkinson M D, Dumontier M, Aalbersberg I J, Appleton G, Axton M, Baak A, Blomberg N, Boiten J-W, da Silva Santos L B, Bourne P E (2016). Sci Data.

[34] (2025). International Union of Crystallography (IUCr) checkCIF service.

[35] (2025). CCDC provide enCIFer as part of (free) Mercury software, available from.

[36] Parks N A, Fischer T G, Blankenburg C, Scalfani V F, McEwen L R, Herres-Pawlis S, Neumann S (2023). Pure Appl Chem.

[37] (2022). R Core Team, RStudio.

[R38] 38FAIR variables were partly adapted from: Le, Y.; Ahlquvist, G. P. ‘Preparing your chemical data for publishing and FAIR sharing’, checklist designed for a workshop at MIT Libraries (Copyright © MASSACHUSETTS INSTITUTE OF TECHNOLOGY). http://bit.ly/FAIRChem210211 (Accessed Jan 7, 2025).

[39] (2025). IUPAC CPEP Subcommittee on Electronic Data Standards.

[40] (2025). Figshare.

[41] (2025). Zenodo.

[42] (2025). GitHub.

[43] (2025). Jupyter.

[44] McDonald R S, Wilks P A (1988). Appl Spectrosc.

[45] (2025). Open Babel: The Open-Source Chemistry Toolbox.

[46] (2025). CC BY-NC 4.0 DEED, Creative Commons.

[47] (2025). CC0 1.0 DEED, Creative Commons.

[48] Hrynaszkiewicz I, Simons N, Hussain A, Grant R, Goudie S (2020). Data Sci J.

[49] Groom C R, Bruno I J, Lightfoot M P, Ward S C (2016). Acta Crystallogr, Sect B: Struct Sci, Cryst Eng Mater.

[50] (2025). Welcome to InChI.

[51] (2025). OpenSMILES.

[52] (2025). nmrXiv.

[53] (2025). NP-MRD.

[54] (2025). BMRB.

[55] (2025). nmrshiftdb2.

[56] David R, Mabile L, Specht A, Stryeck S, Thomsen M, Yahia M, Jonquet C, Dollé L, Jacob D, Bailo D (2020). Data Sci J.

[57] Stall S, Yarmey L, Cutcher-Gershenfeld J, Hanson B, Lehnert K, Nosek B, Parsons M, Robinson E, Wyborn L (2019). Nature.

[58] Hanson R M, Jeannerat D, Archibald M, Bruno I J, Chalk S J, Davies A N, Lancashire R J, Lang J, Rzepa H S (2022). Pure Appl Chem.

[59] Neumann S, Andres A-C, Bach F, Bender T, Bonatto Minella C, Eberl F, Fischer T G, Golub B, Harivyasi S S, Herres-Pawlis S (2024). Res Ideas Outcomes.

[60] (2025). Unified Data Model Pistoia Alliance.

[61] (2025). GO FAIR.

[62] (2025). CODATA.

[63] (2025). Research Data Alliance.

[64] Soiland-Reyes S, Sefton P, Crosas M, Castro L J, Coppens F, Fernández J M, Garijo D, Grüning B, La Rosa M, Leo S (2022). Data Sci.

[65] (2025). A data package is available that contains both human and machine-readable data related to this work.

[66] (2025). DMPOnline.

[67] Lin D, Crabtree J, Dillo I, Downs R R, Edmunds R, Giaretta D, De Giusti M, L’Hours H, Hugo W, Jenkyns R (2020). Sci Data.

[68] (2025). Open Science Framework.

[69] (2025). DRYAD.

[70] (2025). Mendeley Data.

[71] (2025). MassBank Europe.

[72] (2025). MassBank of North America (MoNA).

[73] (2025). IUPAC CPEP Subcommittee on Electronic Data Standards.

[74] Rauh D, Blankenburg C, Fischer T G, Jung N, Kuhn S, Schatzschneider U, Schulze T, Neumann S (2022). Pure Appl Chem.

[75] Maloney M P, Coley C W, Genheden S, Carson N, Helquist P, Norrby P-O, Wiest O (2023). Org Lett.

[76] Mercado R, Kearnes S M, Coley C W (2023). J Chem Inf Model.

