# Supplementary material for: Data accessibility in the chemical sciences: an analysis of recent practice in organic chemistry journals
Source: Beilstein J Org Chem. 2025 May 2;21:864–76. doi: 10.3762/bjoc.21.70 (PMC12051459; doi:10.3762/bjoc.21.70)
Supplement: File 1 — Criteria for journal and article selection, R code for journal sampling, article assessment criteria, files included in the supporting data package, advice for creating a README file. [file Beilstein_J_Org_Chem-21-864-s001.pdf]

BEILSTEIN JOURNAL OF ORGANIC CHEMISTRY

Supporting Information

for

Data accessibility in the chemical sciences: an analysis of recent practice in organic chemistry journals

Sally Bloodworth, Cerys Willoughby and Simon J. Coles

Beilstein J. Org. Chem. **2025,** *21,* 864–876. doi:10.3762/bjoc.21.70

Criteria for journal and article selection, R code for journal sampling, article assessment criteria, files included in the supporting data package, advice for creating a README file

License and Terms: This is a supporting information file under the terms of the Creative Commons Attribution License (https://creativecommons.org/licenses/by/4.0). Please note that the reuse, redistribution and reproduction in particular requires that the author(s) and source are credited and that individual graphics may be subject to special legal provisions.
The license is subject to the *Beilstein Journal of Organic Chemistry* terms and conditions: (https://www.beilstein-journals.org/bjoc/terms)

Criteria for selection of the journals and articles

Specialist, organic chemistry journals were selected from Clarivate™ Journal Citation Reports (JCR)[1] by group (Chemistry) and category (Organic). The resulting 57 journals included in the Science Citation Index Expanded (SCIE) were ranked by (i) five-year impact factor and (ii) total citations, as measures of the journal status. The 25 top-ranked journals in each group were compared and filtered to include only those journals common to both, resulting in a list of 18 journals with status as a top-25 ranked journal by both total citations, and five-year impact factor; these are tabulated in Table S1.

***Table S1**: Specialist organic chemistry journals common to Clarivate™ JCR lists of the 25 top-ranked journals by (i) five-year impact factor and (ii) total citations. The data correspond to JCR year 2021.*
[1]Journals are ranked by five-year impact factor in this table. [2]The 5-year journal Impact Factor is the average number of times articles from the journal published in the past five years have been cited in the JCR year. [3]Total citations are the number of times that a journal has been cited by all journals included in the database in the JCR year.

Rank[1]	Journal title	Five-year Impact Factor[2]	Total citations[3]
1	Natural Product Reports	14.513	14,564
2	Carbohydrate Polymers	9.964	123,307
3	Biomacromolecules	7.055	46,965
4	Organic Letters	5.592	111,922
5	Bioorganic Chemistry	5.321	15,864
6	Advanced Synthesis & Catalysis	5.302	29,229
7	Bioconjugate Chemistry	5.247	19,625
8	Organic Chemistry Frontiers	5.011	13,267
9	Journal of Organic Chemistry	4.031	102,455
10	Organic Process Research & Development	3.762	10,273
11	Organometallics	3.541	36,402
12	Organic & Biomolecular Chemistry	3.464	39,972
13	Bioorganic & Medicinal Chemistry	3.364	33,801
14	European Journal of Organic Chemistry	2.916	27,922
15	Beilstein Journal of Organic Chemistry	2.852	7,756
16	Bioorganic &Medicinal Chemistry Letters	2.774	42,444
17	Synthesis-Stuttgart	2.707	17,983
18	Carbohydrate Research	2.535	17,051

As the attribution of journals within categories of JCR is necessarily subjective, the titles included in any given category are broad and those that straddle sub-disciplines of chemistry are allocated to multiple categories. For example, *Organometallics* appears in both the 'Organic' and 'Inorganic & Nuclear' Chemistry categories, while *Nat. Prod. Rep.* is allocated to the 'Organic' category, and also to 'Biochemistry & Molecular Biology'. The sampling unit for this work is the journal article, with each article evaluated manually. To maximise the sample size within each journal whilst restricting the overall study to a manageable level, the journal titles were refined to a smaller number. So, four journals in the disciplines of biopolymers and biomacromolecules were removed (*Carbohydr. Res.*, *Carbohydr. Polym.*, *Bioconjugate Chem.*, and *Biomacromolecules*), as these are best described as biochemistry journals. Next, the inclusion of two journals with identical aims and scope, *Bioorg. Med. Chem.*, and *Bioorg. Med. Chem. Lett.*, is an unnecessary duplication so the lower ranked title (*Bioorg. Med. Chem. Lett.*) was also removed. Finally, our scope was a report of current practice in the publication of original research results (data), so the review journal *Nat. Prod. Rep.* was also removed.

The 12 remaining journals retain a broad scope around the central discipline of synthesis, catalysis, and methods development in organic chemistry, overlapping with the medicinal, bioorganic, organometallic and process chemistry fields.

Table S2: Specialist organic chemistry journals selected for this study. All titles are common to Clarivate™ JCR lists of the 25 top-ranked journals by (i) five-year impact factor and (ii) total citations (2021). *[1]The study code is an identifier assigned to each journal title for this work. [2]Abbreviations: ACS, American Chemical Society; RSC, The Royal Society of Chemistry.*

Study code[1]	Journal title	Publisher[2]	Issues/year
ADSC	Advanced Synthesis & Catalysis	Wiley-VCH Verlag GMBH	24
BEIL	Beilstein Journal of Organic Chemistry	Beilstein-Institut	1
BIOR	Bioorganic Chemistry	Elsevier	12
BMED	Bioorganic & Medicinal Chemistry	Elsevier	24
EJOC	European Journal of Organic Chemistry	Wiley-VCH Verlag GMBH	48
JORG	Journal of Organic Chemistry	ACS	24
OBIO	Organic & Biomolecular Chemistry	RSC	48
OFRO	Organic Chemistry Frontiers	RSC	24
ORGL	Organic Letters	ACS	24
OPRD	Organic Process Research & Development	ACS	6
OMET	Organometallics	ACS	24
SYNT	Synthesis-Stuttgart	Thieme	24

The data publication requirements for each of the journal titles listed in Table S2, were documented on 01 February 2023, by download of all author instructions and collection of screenshots for those guidelines that were available only as a web resource. For each journal, these instructions describe their policy for data provision by authors, and the data typical of these publications falls in the following groups: (i) spectroscopic data (NMR, IR, UV–vis, Raman, circular dichroism spectroscopies, and mass spectrometry), (ii) chromatography (GC, HPLC, SEC), (iii) thermochemical and physical data (m.p., b.p., elemental analysis, optical rotation), (iv) computational data (in silico theory, code used for analysis or software development), (v) structure information, (vi) cell culture or bioassay data, (vii) crystallographic data.

To address our research questions, sampling of journal articles in a 2-month window of 01 Feb – 31 Mar 2023 was carried out as follows:
A glossary of 'article types' defined by each journal as constituting original research was compiled and is available in the accompanying data package (Article_types.CSV in the CSV files folder). For each journal, a vector of integers was generated in RStudio[2], corresponding to the total number of original research articles published in 2 months, excluding review articles. From the vector for each journal, random sampling in R was used to select 20 articles, i.e., by selection of 20 random integers and matching of these to a chronological list of the articles. The codes used for the journal titles in the transcript match those defined in Table 2.

R Transcript

```
# Select 20 integers from 61, corresponding to 61 articles in ADSC
sample.int(20, n = 61, replace = FALSE)
## [1] 17 36 32 7 34 21 16 58 13 48 40 20 38 50 11 37 4 5 27 49

# Select 20 integers from 86, corresponding to 86 articles in BIOR
sample.int(20, n = 86, replace = FALSE)
## [1] 15 50 74 43 60 27 62 83 72 17 4 19 35 12 32 65 20 56 45 41

# Select 20 integers from 33, corresponding to 33 articles in BMED
sample.int(20, n = 33, replace = FALSE)
## [1] 24 7 14 33 10 23 21 4 8 2 15 19 3 28 25 16 11 1 6 27

# Select 20 integers from 92, corresponding to 92 articles in EJOC
sample.int(20, n = 92, replace = FALSE)
## [1] 3 90 86 30 29 92 44 9 75 78 63 45 14 62 48 88 1 79 37 65
```

```r
# Select 20 integers from 257, corresponding to 257 articles in JORG
sample.int(20, n = 257, replace = FALSE)
## [1] 95 148 69 255 132 131 62 116 244 19 196 163 204 161 246 113 112 249 215
247

# Select 20 integers from 168, corresponding to 168 articles in OBIO
sample.int(20, n = 168, replace = FALSE)
## [1] 124 27 36 140 92 109 149 102 3 150 101 87 39 65 128 138 56 113 4 45

# Select 20 integers from 112, corresponding to 112 articles in OFRO
sample.int(20, n = 112, replace = FALSE)
## [1] 106 23 103 5 74 35 95 94 1 65 41 22 77 110 59 46 31 73 78 76
# Select 20 integers from 291, corresponding to 291 articles in ORGL
sample.int(20, n = 291, replace = FALSE)
## [1] 143 32 124 25 204 176 99 75 251 96 123 97 40 37 52 185 171 100 129 276

# Select 20 integers from 25, corresponding to 25 articles in OPRD
sample.int(20, n = 25, replace = FALSE)
## [1] 25 12 24 14 15 18 19 23 16 2 3 6 11 20 1 9 4 8 7 5

# Select 20 integers from 29, corresponding to 29 articles in OMET
sample.int(20, n = 29, replace = FALSE)
## [1] 29 5 20 16 18 1 28 22 4 26 3 19 10 21 8 15 6 17 2 27

# Select 20 integers from 47, corresponding to 47 articles in SYNT
sample.int(20, n = 47, replace = FALSE)
## [1] 14 18 39 23 28 8 4 30 42 26 27 2 43 11 35 10 6 24 44 33
```

Assessment of the articles

Lastly, assessment of the data objects associated with the selected articles was carried out. The inclusion of 18 possible data types generated in each research article were recorded using Yes = ‘Y’/No = ‘N’ binary responses. Each research article was then assessed against 9 independent categorical variables that describe the main features of the published paper and its associated data, and against 17 ‘FAIR’ variables that measure the extent to which the data associated with the paper meet FAIR data publication standards[3]. These FAIR variables are defined in the accompanying data package (in the FAIR variable coding sheet within FAIR_Practice_Analysis.XLSX and in machine-readable form in FAIR_variable_coding.CSV in the CSV files folder)[4]. The coding of responses for all variables, list of data types associated with each article, and the resulting main dataset from assessment of 240 research papers are described in in the Main dataset sheet within FAIR_Practice_Analysis.XLSX [4] and in machine-readable form in Main_dataset.CSV in the CSV files folder. As all research articles include results based on original (raw) data, and all articles include previously unreported chemical structures, every article was assigned a response to the questions defined in Find_1, Find_4, and Access_3. Then, all remaining questions are assessed only for those studies where primary data has been shared, as established by Access_3.

Data package contents

A data package that contains both human and machine-readable data has been deposited in Zenodo, see https://doi.org/10.5281/zenodo.11068278. The main dataset is available in the Excel file FAIR_Practice_Analysis.XLSX, the contents of which can also be found in machine-readable files: Main_dataset.CSV, Data_types.CSV, and Article_selection.CSV. Explanations of the variable coding used in the studies are in Variable_names.CSV, Codes.CSV, and FAIR_variable_coding.CSV. The R code used

for the article selection can be found in Article_selection.R. Data about article types from the journals that contain original research data is in Article_types.CSV. Additional data were collected to examine other areas of practice and can be found in Extended_Adherence.CSV, Extended_Crystallography.CSV, Extended_DAS.CSV, Extended_File_Types.CSV, and Extended_Submission_Process.CSV. A full list of files in the data package and a short description for each is given in README.TXT.

Creating a README file

The purpose of the README file is to provide a clear and concise description of the content and structure of data included in a dataset, so that the data can be understood and effectively used by others. The following minimum information should be included in the README:

- **Data provenance**: describing who produced the data including persons involved in data collection analysis, and deposition; project title, instruments, methods, experimental conditions, how the data have subsequently been transformed, converted or analysed.
- **Data structure**: how the files have been organised and named, file formats.
- **Code and parameters**: details of scripts and software that have been used to produce or manipulate the data; settings or parameters that are required to replicate/use the data, instructions on how to install and run code; any known issues with the data or code.
- **Sharing and usage**: instructions on how the data can be re-used or validated, license conditions and how the data can be cited, citations for original data if the data set is derived from existing data.
- **Metadata**: Title of the dataset, dates of data collection, dataset versions, date(s) of update(s), keywords, attributes of the dataset (e.g., column and row labels), ORCiD IDs, and funding information.

To make the README machine-readable, avoid proprietary formats, PDF, Word documents and Rich Text Format, and instead use plain Text ('.TXT') or a machine-readable format such as Markdown('.MD')[5]. Cornell Data Services provide a template README file that can be customised together with additional guidance on writing a README with emphasis on metadata[6, 7].

References

[1] Clarivate™ Journal Citation reports: last updated 19 October 2022. https://jcr.clarivate.com/jcr/home. (Accessed January 2023).

[2] R Core Team, RStudio version 2022.12.0+353, R Foundation for Statistical Computing, Vienna, Austria, 2022. https://www.R-project.org/ (Accessed May 2023).

[3] FAIR variables were partly adapted from: Y. Le and G. P. Ahlquvist, *Preparing your chemical data for publishing and FAIR sharing* checklist designed for a workshop at MIT Libraries (Copyright © MASSACHUSETTS INSTITUTE OF TECHNOLOGY). http://bit.ly/FAIRChem210211 (Accessed October 2023).

[4] C. Willoughby, S. Bloodworth and S.J. Coles. 2024, DATASET: Data accessibility in the chemical sciences: an analysis of recent practice in organic chemistry journals. https://doi.org/10.5281/zenodo.11068278.

[5] Markdown Guide, https://www.markdownguide.org/ (Accessed October 2024).

[6] Guide to writing "README" style metadata – Cornell Data Services, https://data.research.cornell.edu/data-management/sharing/readme/ (Accessed March 2024).

[7] AUTHOR_DATASET_ReadmeTemplate.txt, https://cornell.app.box.com/v/ReadmeTemplate (Accessed October 2024).
